# Mandibular Actinomycosis Disguised as a Periapical Lesion

**DOI:** 10.7759/cureus.28002

**Published:** 2022-08-14

**Authors:** Shalini Nair, Shahnaz Mahaboob, Vikas Kuruvilla, Monicah Roy, Shereefa Fareesh

**Affiliations:** 1 Oral and Maxillofacial Pathology, PSM College of Dental Sciences & Research, Thrissur, IND; 2 Oral and Maxillofacial Surgery, PSM College of Dental Sciences & Research, Thrissur, IND

**Keywords:** splendore heoppli phenomenon, sulfur granules, actinomyctes, periapical actinomycosis, actinomycosis cervicofacial

## Abstract

*Actinomycosis* is a rare chronic suppurative granulomatous disease caused by commensal bacteria of Actinomyces species. We report an unusual case of a 20-year-old male patient presenting with pain and swelling to the right lower posterior teeth for a few months. Radiographs revealed a well-defined osteolytic lesion extending from the periapical region of the right mandibular first premolar to the right mandibular second molar. Based on the clinical history of symptoms, a presumptive diagnosis of more commonly appearing jaw lesions like odontogenic cyst/tumor was made. Incision biopsy resulted in an unexpected diagnosis of Actinomycosis, confirmed with Gram stain and Gomori's Methenamine Silver stain. The patient was treated with long-term antibiotics, and follow-up showed a positive response. This article emphasizes the role of histopathology in avoiding the misdiagnosis of such cases.

## Introduction

Actinomycosis, a non-contagious, subacute to chronic bacterial disease, was first described in humans by the German Surgeon James Adolf Israel in 1878. The causative bacteria belong to the Actinomyces spp. and are Gram-positive, non-motile, non-spore-forming, non-acid fast, anaerobic to microaerophilic filamentous rods [1]. The microorganism is a normal commensal of the oral cavity, gastrointestinal tract, and genitourinary tract. The journey from a low virulent, peacefully coexisting organism to a deceptive, destructive one is facilitated by many factors such as a breach in the integrity of the mucosal barrier, presence of devitalized tissue, and synergistic action of co-pathogens [2].

Actinomycosis is characterized by suppuration, contiguous spread often defying tissue planes, formation of multiple draining sinuses, invasion into deeper tissues, and later granulomatous inflammation and fibrosis. It has been referred to as a 'mimicker,' 'pretender,' and 'imitator' because of its variable clinical presentation and elusive diagnosis. It also holds in our case, where a young, healthy individual presented with a periapical lesion with no characteristic features of actinomycotic infection and no apparent risk factors. The diagnosis was possible only after histopathologic examination, emphasizing its role in diagnosing clinically misleading lesions correctly. To the best of our knowledge, there's only one reported case of periapical Actinomycosis associated with a vital tooth [3].

## Case presentation

A 20-year-old male patient reported to a Dental college in India in 2020 with pain and swelling about the right mandibular molar- premolar region for six months.

The patient had initially consulted a general Dental practitioner and had undergone treatment with broad-spectrum antibiotics which resulted in temporary relief from pain. When the swelling persisted, he reported to our institution. He had a history of intermittent fever. He did not recall any episode of recent facial trauma, dental procedures, or dental caries. His oral hygiene and periodontal health were reasonable. His medical history was non-contributory. On extraoral examination, there was mild asymmetry on the right side of the face. Intraoral examination revealed a firm swelling with buccal cortical plate expansion to the lower right premolar molar region. No tenderness on percussion was elicited in any of the associated teeth. The teeth in the vicinity normally responded to electric vitality testing. There were no draining sinuses or dental foci of infection.

**Figure 1:**
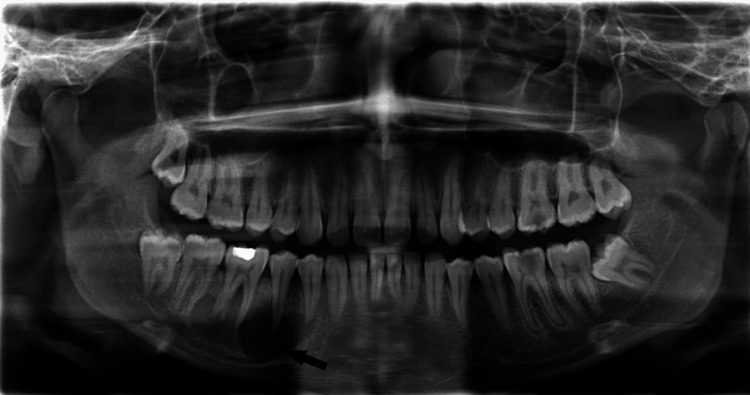
OPG showing a well-defined radiolucent periapical lesion associated with right mandibular premolars and first molar. OPG- Orthopantamogram

Orthopantamogram (OPG) showed a well-defined osteolytic lesion of varying radiodensity extending from the periapical region of the right mandibular first premolar to the second molar (Fig 1).

**Figure 2:**
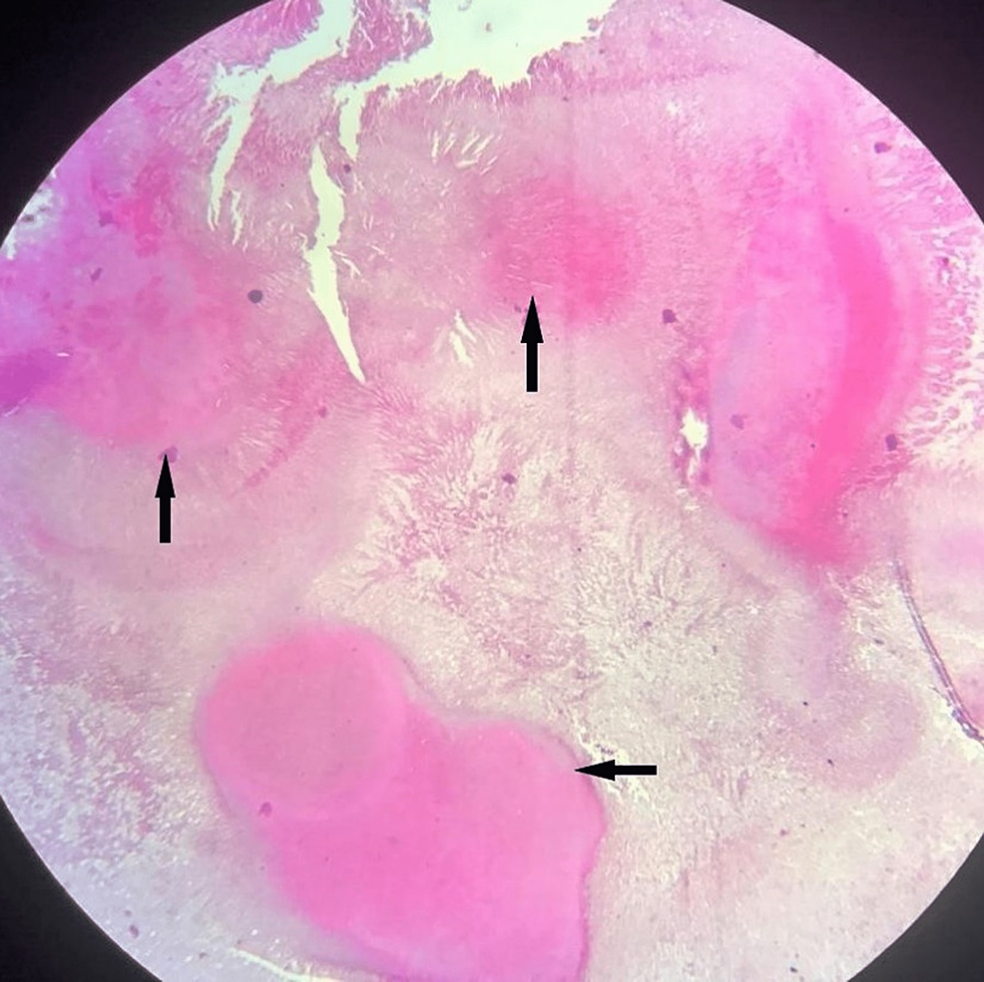
Colonies of filamentous bacteria in a necrotic background (H&E, 10x)

**Figure 3:**
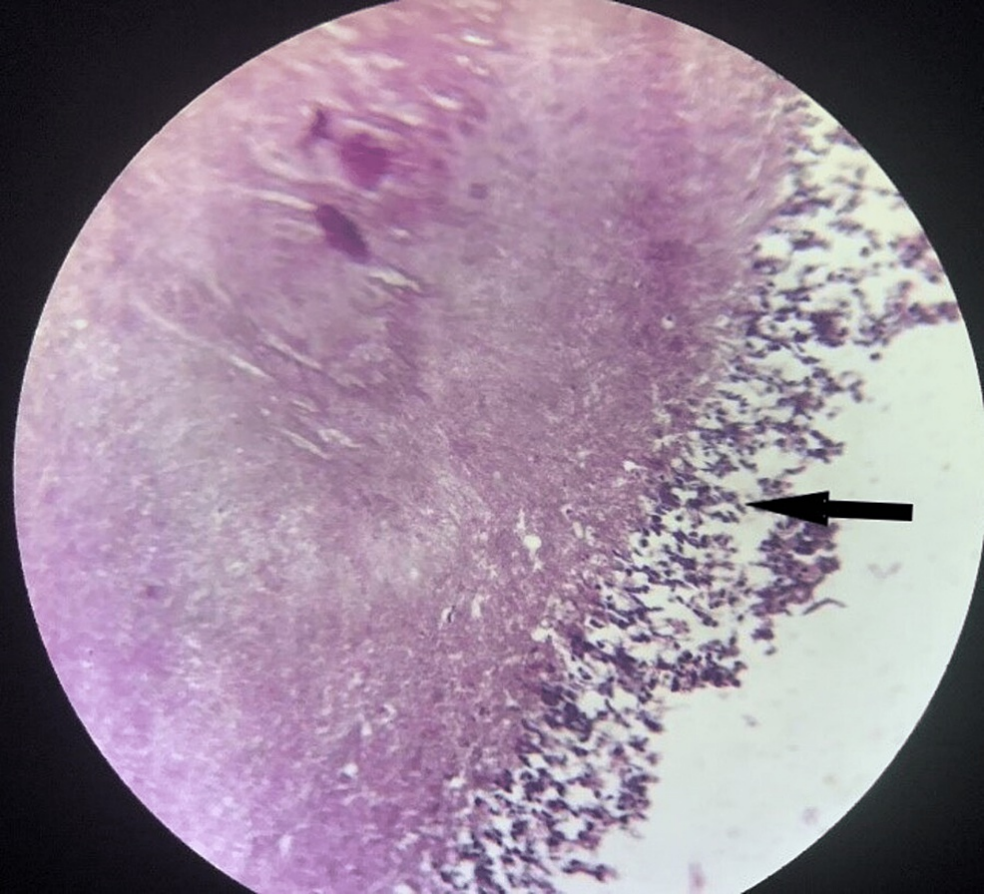
Sulfur granule surrounded by numerous polymorphonuclear leukocytes. (H&E, 10x)

Based on the clinical and radiographic evaluation, a provisional diagnosis of an odontogenic cyst or unicystic ameloblastoma was given. The patient underwent a surgical intervention under local anesthesia. A full thickness mucoperiosteal flap was elevated, and the defect was curetted. The tissue obtained was sent for histopathological examination. Histopathology with H & E stain showed multiple fragments of connective tissue with extensive chronic inflammation and foci of necrotic acellular areas. Clumps of basophilic filamentous bacteria in a vaguely rosette-like configuration surrounded by chronic inflammatory cells were observed (Fig 2, 3). There were no histopathological features of a cystic lesion.

**Figure 4:**
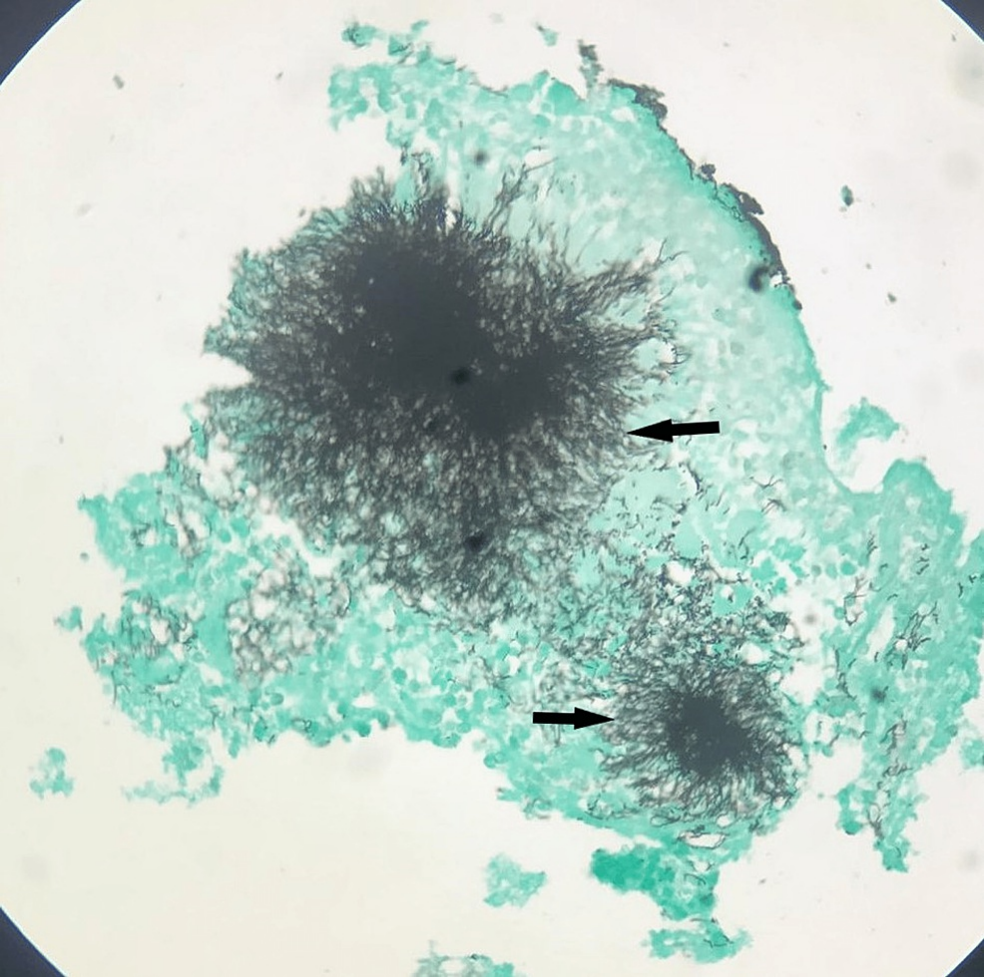
Bacterial colonies with radiating hyphae. (Gomori methanamine-silver nitrate stain, 10x)

Based on these features, Actinomycosis was suspected. After that, Gomori methenamine-silver nitrate (GMS) stain and Grams staining were done, which demonstrated dense clusters of radiating, branching filamentous bacteria confirming the diagnosis of periapical Actinomycosis (Fig 4).

**Figure 5:**
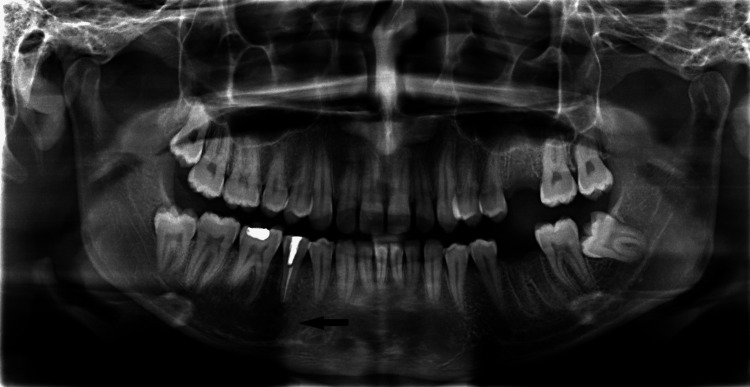
OPG on follow-up after 6 months showing resolution of the lesion

The patient was treated with a high dose of intravenous penicillin G (8 million U per day) for two weeks, followed by oral Penicillin (500mg q.i.d) for four weeks. An intentional root canal treatment (RCT) was also done for the mandibular second premolar associated with the lesion. A six-month follow-up radiograph showed the radiolucent defect's resolution (Fig 5).

## Discussion

Periapical presentation of Actinomycosis is sporadic. The portal of entry of the microorganisms is usually trauma, fractures, invasive dental procedures, hematogenous, infected root canals, and maybe periodontal pockets [4]. Most of the reported cases of periapical Actinomycosis were associated with non-vital or root canal-treated teeth. The instigating factor for infection in our case is unclear. However, it could be postulated as a polymicrobial infection; the companion bacteria increase the virulence of Actinomyces by elaborating toxins, tissue degrading enzymes such as hyaluronidase, or decreasing the oxygen tension in tissues [1].

The most common clinical presentation of actinomycotic infection is chronic mass, which may be suppurative or indurated and can be associated with discharging sinuses. Karanfilian KM et al. proposed a classification of three stages for cervicofacial Actinomycosis: localized infection without sinus involvement, localized infection with sinus involvement, and disseminated infection [5]. Purulent discharge from the sinuses can be examined for bacterial colonies (sulfur granules), which present as cauliflower-shaped basophilic clusters with radiating hyphae tipped with eosinophilic clubs in the background of inflammatory cells [6]. The eosinophilic periphery is due to the Splendore Heoppli phenomenon, a deposition of club or star-shaped amorphous, eosinophilic material around microorganisms or inert substances [7]. Sulfur granules are not pathognomonic, as they are only present in 35-55% of cases and are also seen with Nocardia, Streptomyces, and Peptostreptococcus [8,9].

This case is unusual as it lacked the typical clinical features of actinomycotic osteomyelitis, such as multiple sinuses with a discharge of pus containing sulfur granules, leading to delayed diagnosis and management of the infection. Cho et al. have reported a similar case of mandibular Actinomycosis in a pediatric patient who presented with non-specific symptoms and no predisposing factors [3]. Such cases, even though rare, warrant the inclusion of Actinomycosis in the differential diagnosis of mandibular pain and swelling.

Actinomyces israelli responds well to antibiotics such as Penicillin, sulphonamides, streptomycin, tetracyclines, erythromycin, and rifampicin, but high doses of long-term Penicillin are the treatment of choice. The use of intracanal irrigants and medicaments in eradicating the infection has also been reported [10]. In this case, the patient was given a long-term course of Penicillin. Also, a vital tooth RCT was done on the involved premolar anticipating pulpal death post-surgically. A satisfactory response to the above treatment strategy was observed on follow-up.

## Conclusions

Actinomycotic infection of the oral and maxillofacial region poses a challenge in diagnosis due to its multifaceted and non-specific clinical presentation. This case is unusual because the absence of classic symptoms leads to misdiagnosis and a lack of obvious etiological factors for infection in an immunocompetent individual. Also, there is a paucity of reports of similar cases of periapical Actinomycosis associated with vital teeth. Actinomycosis being an elusive diagnosis due to its varied presentation, the importance of histopathological examination of every bit of tissue obtained therapeutically or diagnostically is reiterated in the above case.
